# Tumor-Specific Hsp70 Plasma Membrane Localization Is Enabled by the Glycosphingolipid Gb3

**DOI:** 10.1371/journal.pone.0001925

**Published:** 2008-04-02

**Authors:** Mathias Gehrmann, Gerhard Liebisch, Gerd Schmitz, Robin Anderson, Claudia Steinem, Antonio De Maio, Graham Pockley, Gabriele Multhoff

**Affiliations:** 1 Department of Radiotherapy and Radiooncology, Klinikum rechts der Isar, Technische Universität München, Munich, Germany; 2 Institute for Clinical Chemistry, University Hospital Regensburg, Regensburg, Germany; 3 Peter MacCallum Cancer Institute, East Melbourne, Victoria, Australia; 4 Institute for Organic and Biomolecular Chemistry, Georg-August University, Göttingen, Germany; 5 Department of Surgery, University of California San Diego, La Jolla, California, United States of America; 6 School of Medicine and Biomedical Sciences, University of Sheffield, Sheffield, United Kingdom; 7 Institute of Pathology, German Research Center for Environmental Health (GmbH), Helmholtz Center Munich, Munich, Germany; University of Helsinki, Finland

## Abstract

**Background:**

Human tumors differ from normal tissues in their capacity to present Hsp70, the major stress-inducible member of the HSP70 family, on their plasma membrane. Membrane Hsp70 has been found to serve as a prognostic indicator of overall patient survival in leukemia, lower rectal and non small cell lung carcinomas. Why tumors, but not normal cells, present Hsp70 on their cell surface and the impact of membrane Hsp70 on cancer progression remains to be elucidated.

**Methodology/Principal Findings:**

Although Hsp70 has been reported to be associated with cholesterol rich microdomains (CRMs), the partner in the plasma membrane with which Hsp70 interacts has yet to be identified. Herein, global lipid profiling demonstrates that Hsp70 membrane-positive tumors differ from their membrane-negative counterparts by containing significantly higher amounts of globotriaoslyceramide (Gb3), but not of other lipids such as lactosylceramide (LacCer), dodecasaccharideceramide (DoCer), galactosylceramide (GalCer), ceramide (Cer), or the ganglioside GM1. Apart from germinal center B cells, normal tissues are Gb3 membrane-negative. Co-localization of Hsp70 and Gb3 was selectively determined in Gb3 membrane-positive tumor cells, and these cells were also shown to bind soluble Hsp70-FITC protein from outside in a concentration-dependent manner. Given that the latter interaction can be blocked by a Gb3-specific antibody, and that the depletion of globotriaosides from tumors reduces the amount of membrane-bound Hsp70, we propose that Gb3 is a binding partner for Hsp70. The *in vitro* finding that Hsp70 predominantly binds to artificial liposomes containing Gb3 (PC/SM/Chol/Gb3, 17/45/33/5) confirms that Gb3 is an interaction partner for Hsp70.

**Conclusions/Significance:**

These data indicate that the presence of Gb3 enables anchorage of Hsp70 in the plasma membrane of tumors and thus they might explain tumor-specific membrane localization of Hsp70.

## Introduction

Heat shock proteins (HSPs) with a molecular weight of approximately 70 kDa are located in most cellular compartments, in which they support the folding of nascent polypeptides, prevent protein aggregation and assist the transport of other proteins across membranes [1], [2]. Using flow cytometry of viable tumor cells and selective cell surface iodination, we have previously demonstrated a tumor-specific, plasma membrane localization of Hsp70, the major stress-inducible member of the HSP70 family [3]. These findings concur with other studies that have used global profiling of membrane-bound proteins to show that there is an abundance of molecular chaperones, including Hsp70, in the plasma membrane of tumor cell lines [4]. Extracellular and membrane-bound Hsp70 play pivotal roles in the innate immune system [5] and we have shown that the latter acts as a target structure for natural killer (NK) cells. Using autologous tumor sublines which differentially express membrane Hsp70, as generated by antibody-based cell sorting, we have demonstrated that Hsp70 membrane-positive tumor cells are significantly more susceptible to NK cell-mediated killing than their counterparts which express low levels of membrane-bound Hsp70 [3], [5]. Although the immunological relevance of membrane-bound Hsp70 is apparent, little is known about the binding partners that enable membrane anchorage of Hsp70 in viable tumor cells.

Herein we report that approximately 15 to 20% of the total cellular Hsp70 content of tumor cells is present on the cell surface. High-salt conditions and low/high pH did not alter Hsp70 membrane expression (unpublished observation), a finding which indicates that it is unlikely that Hsp70 is bound to a proteinous cell surface component. These data suggest that, in contrast to antigen presenting cells to which HSPs bind via cell surface receptors [6], in tumor cells, Hsp70 might be associated with fatty acids within the plasma membrane, as was suggested by Hightower and Guidon in 1989 [7]. More recently, evidence that stress proteins, including Hsp70, are present in glycosphingolipid and cholesterol-rich microdomains (CRMs) of tumor cells has accumulated [8]–[10]. In line with these findings, we show that the depletion of cholesterol results in loss of membrane-bound Hsp70 in tumor cells. Furthermore, a comparative analysis of the lipid molecular species in the plasma membrane of Hsp70 membrane-positive and -negative tumor cells revealed that the content of globoyltriaosylceramide (Gb3), a component of cholesterol-rich microdomains, but not that of other ceramide-derived glycosphingolipids, was significantly higher in Hsp70 membrane-positive tumor sublines. The globotriaoside Gb3 is a marker for the germinal stage B cell development and thus is highly overexpressed in Burkitt's lymphoma cells such as Daudi cells [11]–[13]. Functionally, Gb3 is a receptor for the AB5-toxin Shiga toxin and related toxins such as Verotoxin [14], [15]. Co-staining of Gb3 and Hsp70 in the plasma membrane of Hsp70-positive tumor cells and a concentration-dependent binding of Hsp70 to Gb3-positive tumors is consistent with this finding. The selective binding of Hsp70 to artificial Gb3-containing lipid vesicles further supports our hypothesis that Gb3 is a prerequisite for the integration of Hsp70 in the plasma membrane in tumor cells.

## Results

### Comparative Analysis of the Hsp70 Content in the Cytosol and on the Plasma Membrane of Tumor Cells

The human colon (CX2) and human pancreas (Colo357) carcinoma cell lines were separated into sublines exhibiting a stably high (CX+, Colo+) or low (CX−, Colo−) membrane Hsp70 expression by Hsp70 monoclonal antibody (mAb)-based cell sorting, as has been described previously [16], [17]. Under physiological conditions, 87±8% of CX+ and 73±5% of Colo+ tumor sublines were Hsp70 membrane-positive, whereas only 23±6% of CX− and 34±5% of Colo− carcinoma sublines were Hsp70 membrane-positive (Table 1).

**Table 1:** Flow cytometric analysis of viable, 7-AAD- and Annexin-V-negative CX+/CX−, Colo+/Colo− and HeLa+/HeLa− (HeLa Bag-4/HeLa neo) carcinoma sublines and Daudi Burkitt's lymphoma cells.

	Proportion of Hsp70 membrane-positive cells (%±SE)^a^						
	CX+	CX−	Colo+	Colo−	HeLa+	HeLa−	Daudi
**Hsp70**	87±8	23±6	73±5	34±5	78±8	36±4	99±4

a Data are mean percentages of Hsp70 (cmHsp70.1) plasma membrane positive cells±SE from 3 independent experiments.

We have previously shown that transfection of the human cervix carcinoma cell line HeLa with Bag-4 increases cell surface expression of Hsp70 (HeLa+, 78±8% vs 36±4% on neo-transfected HeLa cells, HeLa-) [18]. Bag-4/neo transfection was therefore used to generate another tumor cell model in which a differential membrane Hsp70 expression was apparent (Table 1). The Burkitt's lymphoma cell line Daudi displays a strong Hsp70 staining on the plasma membrane of all cells (99±4%).

To exclude non-specific staining, membrane expression was only measured in viable (7-AAD negative) cells which had intact plasma membranes. In addition, phosphatidylserine (PS), an early marker for apoptosis, was not present on the outer leaflet of the plasma membrane, since none of the tumor cell lines bound fluorescently labelled Annexin-V in the presence of physiological concentrations of Ca^2+^ (data not shown).

**Figure 1:**
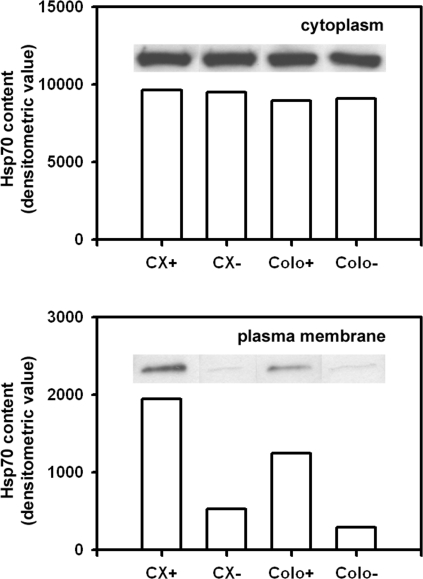
Quantification of cytosolic and plasma membrane-bound Hsp70 in CX+/CX− and Colo+/Colo− carcinoma sublines. Western blot analysis of biotinylated whole cell lysates (cytoplasm, upper graph), and plasma membranes (lower graph) of CX+/CX− and Colo+/Colo− tumor sublines. A corresponding Western blot was stained with the Hsp70 specific mAb cmHsp70.1. The figure shows a representative blot from three independent experiments. The Hsp70 surface phenotypes of the tumor sublines, as determined by flow cytometric analysis, are summarized in Table 1.

The amount of membrane-bound Hsp70 was determined by Western blot analysis of biotinylated plasma membrane proteins (purity >97%). For this, surface-bound proteins on intact tumor cells were selectively biotinylated and labeled proteins were isolated using avidin agarose beads. Bound proteins and whole cell membranes from the same tumor subline were subjected to SDS-PAGE, blotted on nitrocellulose and stained with an Hsp70-specific mAb (cmHsp70.1, multimmune GmbH). As shown in Figure 1, the cytoplasmic Hsp70 content in CX+/CX− and Colo+/Colo− sublines was similar under physiological conditions. We have previously reported that the Hsp70 content is similarly elevated in both tumor cell systems following stress [18]. A comparison of cytosolic and membrane-bound Hsp70 content revealed that approximately 20% and 15% of the total Hsp70 is present in the plasma membrane of CX+ and Colo+ tumor cells, respectively. In contrast, less than 3% of total Hsp70 was found in the plasma membranes of Hsp70 low-expressing CX− and Colo− tumor sublines.

### Hsp70 is associated with cholesterol rich microdomains (CRMs)

The amount of membrane Hsp70 is not affected by high salt or by alkali/acid treatment (unpublished observation). Thus, a protein-mediated interaction of Hsp70 with a protein compound of the cell surface appears to be unlikely. Therefore, it was hypothesized that Hsp70 might be directly associated with lipid components of the plasma membrane. However, a comparative lipid profiling of whole cell membranes and purified plasma membranes of corresponding tumor sublines with differential Hsp70 membrane expression using electrospray tandem mass spectrometry revealed no significant differences in phosphatidylcholine (PC), sphingomyelin (SPM), dihydrosphingomyelin (DiSPM), phosphatidylethanolamine (PE), phosphatidylserine (PS), ceramide (Cer), and glucosylceramide (GluCer) composition (Table 2). Although the amount of the sphingolipids sphingosine (SPH) and sphinganine (SPA) was 100-fold lower than other lipid components, levels in Hsp70 membrane-positive and –negative tumor subtypes were similar (data not shown).

**Table 2:** Lipid composition and lipid ratio of whole cell membranes and purified plasma membranes of CX+/CX−, Colo+/Colo−, and HeLa+/HeLa− tumor sublines.

Lipids	PC	SPM	DihSPM	PE	PS	Cer	GluCer
	Whole cell membranes						
**CX+**	38.8±8.1^a^	2.8±0.5	0.7±0.2	6.2±0.4	6.8±2.7	0.6±0.4	0.1±0.1
**CX−**	37.0±6.7	3.9±0.3	0.5±0.2	5.3±0.6	8.5±4.8	0.6±0.5	0.1±0.1
**Ratio**	**1.04**	**0.72**	**1.4**	**1.17**	**0.80**	**1.0**	**1.0**
**Colo+**	38.9±8.7	5.7±1.2	0.7±0.3	6.9±1.0	8.8±5.1	0.5±0.3	0.1±0.1
**Colo−**	36.7±7.4	5.4±0.5	0.8±0.4	6.7±0.6	8.5±4.1	0.5±0.3	0.1±0.1
**Ratio**	**1.06**	**1.06**	**0.88**	**1.03**	**1.04**	**1.0**	**1.0**
**HeLa+**	31.2±4.7	7.2±0.3	0.6±0.1	9.7±0.4	9.3±3.9	0.4±0.4	0.1±0.1
**HeLa−**	30.3±5.6	7.6±0.5	0.5±0.2	10.7±1.1	8.4±3.2	0.5±0.5	0.1±0.1
**Ratio**	**1.03**	**0.95**	**1.20**	**0.91**	**1.11**	**0.80**	**1.0**

Lipids	PC	SPM	DihSPM	PE	PS	Cer	GluCer
	Plasma membranes						
**CX+**	44.1±0.3	3.3±0.2	0.7±0.0	6.1±0.4	4.2±0.2	0.3±0.1	0.1±0.0
**CX−**	41.1±3.0	3.9±0.4	0.8±0.1	5.6±0.7	4.2±0.2	0.3±0.0	0.1±0.0
**Ratio**	**1.07**	**0.85**	**0.88**	**1.09**	**1.0**	**1.0**	**1.0**
**Colo+**	41.8±0.1	5.1±5.3	1.0±0.0	5.9±0.0	5.0±0.3	0.3±0.0	0.0±0.0
**Colo−**	42.0±0.2	5.3±0.1	0.8±0.0	6.1±0.1	4.8±0.4	0.3±0.0	0.0±0.0
**Ratio**	**0.99**	**0.96**	**1.25**	**0.97**	**1.04**	**1.0**	
**HeLa+**	35.9±1.3	7.5±0.0	0.7±0.0	9.6±0.5	5.4±0.0	0.1±0.0	0.0±0.0
**HeLa−**	35.5±1.2	7.5±0.7	0.7±0.2	10.7±0.7	5.5±0.1	0.1±0.0	0.0±0.0
**Ratio**	**1.01**	**1.01**	**1.0**	**0.90**	**0.98**	**1.0**	

Data are displayed as the lipid content and ratio in Hsp70 membrane-positive vs membrane-negative carcinoma sublines. The calculation for total lipid content is based on the sum of all analyzed lipid classes in nM/mg protein. The lipid classes are calculated from the respective mol % value of all analyzed lipids.

a Data are means±SE of 3 to 4 independent experiments. PC, phosphatidylcholine; SPM, sphingomyelin; DiSPM, DihydroSPM; PE, phosphatidylethanolamine; PS, phosphatidylserine; Cer, ceramide; GluCer, glucosylCer

**Figure 2:**
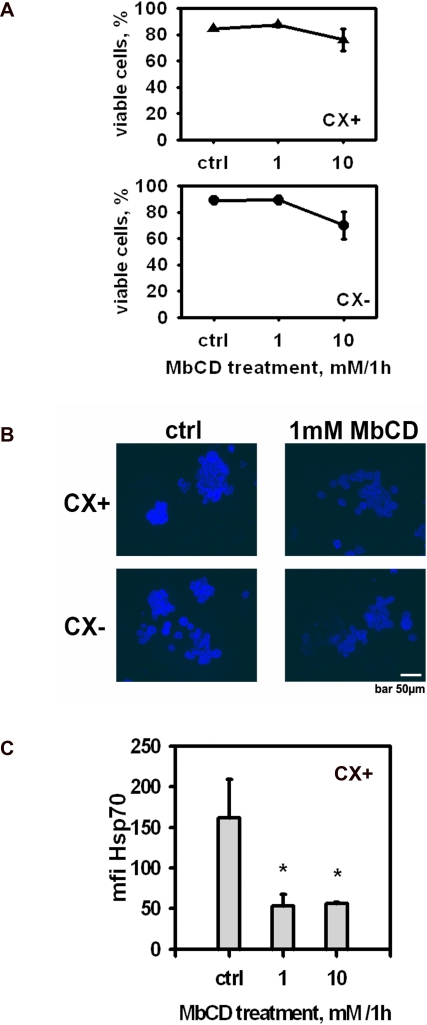
Hsp70 is associated with detergent resistant microdomains (DRMs). (A) Definition of the sublethal concentration of the raft disrupting agent methyl-beta cyclodextrin (MbCD). Following incubation of CX+ and CX− tumor sublines with 1 and 10 mM MbCD for 1 h, cells were washed and viability was determined by trypan blue staining after a 12 h recovery period. (B) The images show filipin III binding to untreated (ctrl, left graphs) and to CX+/CX− tumor sublines treated with the sublethal concentration of 1 mM MbCD (right graphs) for 1 h. (C) Cell surface density of Hsp70 in untreated (ctrl) and MbCD (1 mM, 10 mM) treated CX+ cells. Viability was 97.7±0.4 for control and 95±0.7 for MbCD treated cells. The data represent mean fluorescence intensity (mfi) values of three independent flow cytometric analyses. *Indicates values that are significantly different from control (P = 0.006).

Given work from others which suggests that, apart form the cytosolic localization, Hsp70 can be located in CRMs [6]–[9], the potential association of Hsp70 with CRMs was investigated using CX+ and CX− colon carcinoma sublines that had been treated with reagents which dissociate cholesterol from the plasma membrane. Methyl-beta cyclodextrin (MbCD) concentrations of up to 10 mM were determined to be sublethal using a standard viability assay (Figure 2A). Cholesterol depletion following treatment with 1 mM MbCD was confirmed on the basis of a dramatic reduction in the intensity of filipin III binding in both CX+ and CX− tumor sublines (Figure 2B). Treatment of CX+ tumor cells with 1 and 10 mM MbCD resulted in a significant reduction in the mean fluorescence intensity (mfi) of Hsp70 expression on CX+ cells (P = 0.006; Figure 2C); whereas no change in expression was detected on CX− tumor cells which express low levels of Hsp70 membrane expression (data not shown). Similar results were obtained with Colo+/Colo− tumor sublines (data not shown). Taken together, these findings suggest that Hsp70 might be associated with CRMs in the plasma membrane of tumor cells.

### Globotriaosylceramide (Gb3) is Increased in Hsp70 Membrane-Positive Tumor Cells and Co-Localizes with Hsp70

**Figure 3:**
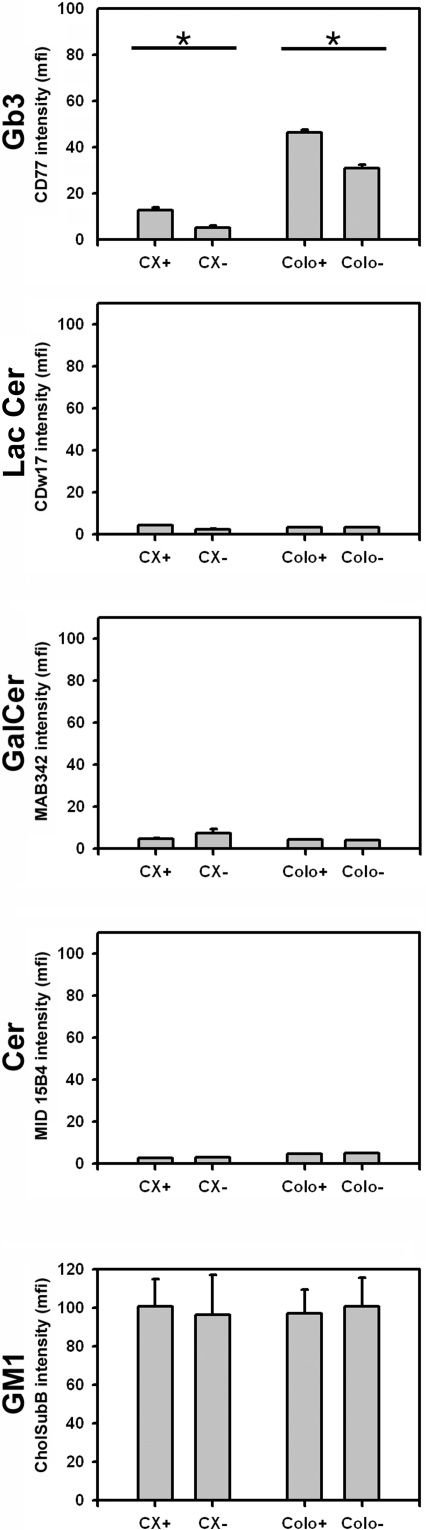
Globotriaosylceramide content is significantly higher in Hsp70 membrane-positive tumor sublines compared to their negative counterparts. Comparative analysis of the cell surface density of glycosphingolipids such as Gb3 (globotriaosylceramide, CD77 Ab), LacCer (lactosylceramide, CDw17 Ab), DoCer (dodecasaccharideceramide, CD65s Ab, data not shown), GalCer (galactosylceramide, MAB 342 Ab), Cer (ceramide, MID15B4 Ab), and GM1 (Cholera Toxin subunit B) in CX+/CX− and Colo+/Colo− tumor sublines. *Indicates values for Hsp70 membrane-positive (CX+, Colo+) and the corresponding Hsp70 membrane-negative (CX−, Colo−) tumor subline that are significantly different (P<0.03).

A comparative analysis of different glycosphingolipids which are components of CRMs (ceramide, GM1) in Hsp70 membrane-positive and -negative tumor sublines revealed that the level of globotriaosylceramide (Gb3/CD77) in CX+ and Colo+ tumor sublines is significantly greater that that in CX− and Colo− tumor sublines (Figure 3). In contrast, levels of lactosylceramide (LacCer/CDw17), galactosylceramide (GalCer/MAB 342), and ceramide (Cer/MID 15B4) were low, and were similar. Although levels of the glycosphingolipid component dodecasaccharideseramide (DoCer/CD65s) in the CX+/CX− tumor subline system were 5-fold higher than in the Colo+/Colo− tumor subline system, no differences were detectable between the Hsp70 membrane-positive and -negative counterparts (data not shown). Therefore, DoCer was also excluded as a potential interaction candidate for membrane Hsp70 interactions.

**Figure 4:**
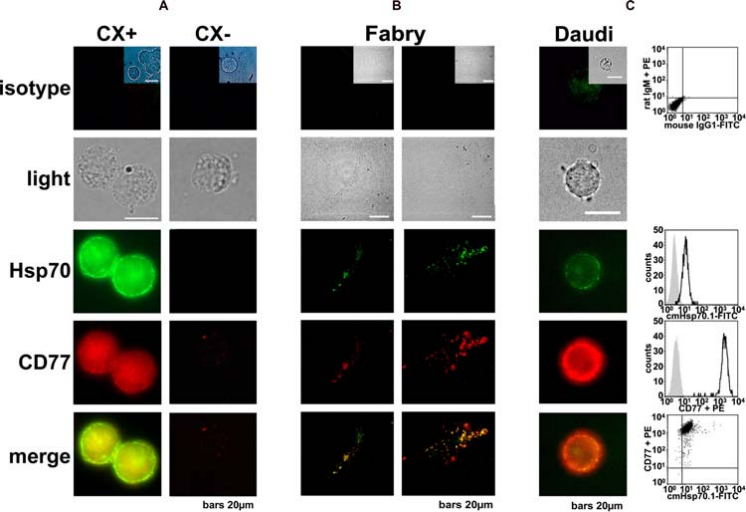
Bright field and fluorescence microscopic analysis of CX+ and CX− tumor sublines (A), Fabry fibroblasts (B), and Daudi Burkitt's lymphoma cells (C). Cells were stained either with a relevant isotype-matched control antibody (isotype) or with an Hsp70 (cmHsp70.1-FITC, green) or Gb3 (CD77 plus Cy3-conjugated secondary antibody, red) specific antibody. The co-localization of Hsp70 and Gb3 is visualized in yellow as a merge of red and green in the lowest panel. Scale bar marks 20 µm. In comparison to CX+ and Fabry cells, the density of Gb3 on the cell membrane of Daudi cells is markedly increased, as indicated by a high mean fluorescence intensity of the CD77 staining. A double stain of Hsp70-FITC and Gb3-Cy3 indicates co-localization of both markers on the cell surface (lower right graph). Similar findings were obtained in experiments using the Colo+/Colo− tumor sublines (data not shown). On the right hand panel of Figure 4C, representative flow cytometric profiles of Daudi cells are shown; the upper graph represents the double staining pattern of the isotype-matched antibodies IgM-PE and IgG1-FITC; the graphs below show single staining of Daudi cells with Hsp70-FITC (second graph) and CD77-PE (third graph). The fourth graph represents the double-staining pattern of Daudi cells using Hsp70-FITC and CD77-PE antibodies.

A merge of the staining images (Hsp70-FITC in green and Gb3 in red, CD77 plus Cy3-conjugated secondary antibody) of cell lines which exhibit differential Gb3 expression, indicated that these moieties co-localize in the plasma membrane of CX+, but not CX− tumor sublines (Figure 4A). Similar data were obtained using fibroblasts derived from Fabry patients (Figure 4B). Fabry disease is a rare X-linked hereditary lipid storage disorder in which the deficiency in the enzyme galactosidase A causes a drastic increase in Gb3 levels. The Burkitt's lymphoma cell line Daudi is extremely positive for Gb3, and Hsp70 and Gb3 also co-localize on the membranes of these cells (Figure 4C).

Furthermore, incubation of Hsp70 membrane-positive CX+ tumor cells with a sublethal dose of 1-Phenyl-2-Hexadecanoyl Amino-3-Morpholino-1-Propanol (PPMP1), which eliminates ceramide-based glycosphingolipids including Gb3 from the plasma membrane, for 3 and 6 days also resulted in a depletion of Hsp70 from the tumor cell surface (Table 3). These findings further support the hypothesis that Gb3 serves as an interaction partner for Hsp70 in CRMs on the plasma membrane of tumor cells and Fabry fibroblasts.

**Table 3:** Flow cytometric analysis of viable, 7-AAD- and Annexin-V-negative CX+ tumor cells either untreated (Ctrl) or after treatment with a sublethal dose of 1 phenyl-2-hexadecanoyl amino-3-morpholino-1-propanol (PPMP1, 25 µM) for 3 or 6 days.

	Density of Hsp70 membrane-positivity (mfi±SE)^a^		
	Ctrl	PPMP1 (d3)	PPMP1 (d6)
**CX+**	16.53±2.7	11.1±1.5	8.46±6.6

Data are displayed as the mean fluorescence intensity of Hsp70 positive cells±SE from at least 3 independent experiments.

a means significantly different from control (P<0.05).

### Globotriaosylceramide (Gb3) is a Prerequisite for an Interaction of Hsp70 with Artificial Raft-Like Liposomes

**Figure 5:**
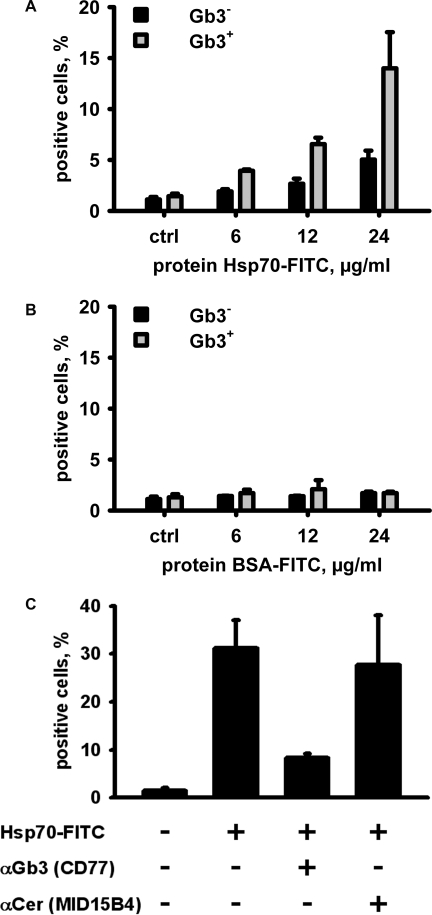
Binding of Hsp70-FITC, and BSA-FITC to viable Gb3 membrane-negative K562 and Gb3 membrane-positive Daudi cells. (A) Significant binding of Hsp70-FITC was observed to Daudi cells at a concentration of 12 µg/ml and 24 µg/ml. (B) BSA-FITC did neither bind to K562 nor to Daudi cells, at any of the tested concentrations. (C) Specific blocking of Hsp70-FITC binding by anti-Gb3 monoclonal antibody CD77. Daudi cells were kept either untreated or incubated with anti-Gb3 (CD77), or anti-ceramide (MID15B4) antibodies (5 µg/ml) for 30 min on ice prior to incubation with Hsp70-FITC (60 µg/ml, 30 min on ice) and analysis by flow cytometry.

**Figure 6:**
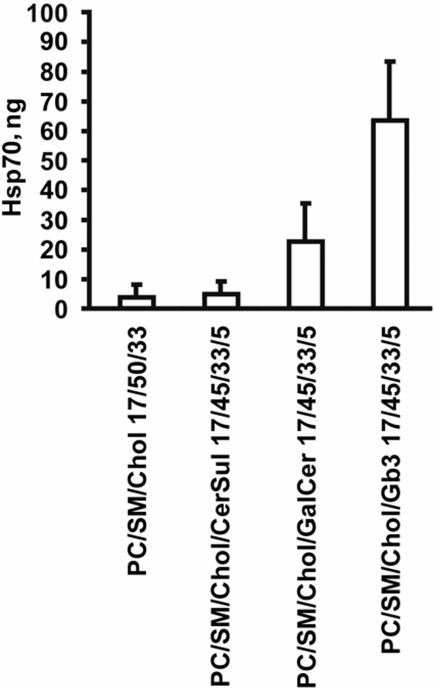
Hsp70 predominantly interacts with artificial raft liposomes containing Gb3. Quantification of the amount of recombinant Hsp70 protein associated with unilamellar raft-like liposomal pellet fraction consisting of 1-palmitoyl-2-oleoyl-sn-glycero-3-phosphocholine (PC)/brain sphingomyelin (SM)/cholesterol (Chol), (PC/SM/Chol, 17/50/33); 1-palmitoyl-2-oleoyl-sn-glycero-3-phosphocholine (PC)/brain sphingomyelin (SM)/cholesterol (Chol)/brain cerebroside sulfatide (CerSul) (PC/SM/Chol/CerSul, 17/45/33/5); 1-palmitoyl-2-oleoyl-sn-glycero-3-phosphocholine (PC)/brain sphingomyelin (SM)/cholesterol (Chol)/galactocerebroside (GalCer) (PC/SM/Chol/GalCer, 17/45/33/5); and 1-palmitoyl-2-oleoyl-sn-glycero-3-phosphocholine (PC)/brain sphingomyelin (SM)/cholesterol (Chol)/globotriasoylceramide (Gb3) (PC/SM/Chol/Gb3, 17/45/33/5). Data are the total amount of Hsp70 (ng) in the pellet fraction containing liposomes and are means (±SE) of 3 to 5 independent experiments.

To further characterize the interaction of Hsp70 with Gb3 in CRMs we performed protein binding studies. Incubation of Gb3-negative (K562) and Gb3-positive (Daudi) cells with different concentrations (6, 12, 24 µg/ml) of Hsp70-FITC revealed a significantly higher binding of Hsp70-FITC to Daudi cells compared to K562 cells at concentrations of 12 and 24 µg/ml (Figure 5A). In contrast, BSA-FITC which was used as a control protein did not bind to Gb3-negative or –positive cells at identical concentrations (Figure 5B). Similar results were seen with CX+ and CX− tumor sublines (data not shown). Blocking studies indicated that the binding of Hsp70-FITC (60 µg/ml) to Daudi cells could be completely inhibited by the Gb3 (CD77)-specific mAb, but not by a control antibody specific for ceramide (MID15B4). These data are in line with the hypothesis that Gb3 serves as an interaction partner for Hsp70 on the cell surface of tumor cells. To further prove this proposition, we performed vesicle copellation assays using physiologically relevant lipid mixtures, including 1-palmitoyl-2-oleoyl-sn-glycero-3-phosphocholine (PC)/brain sphingomyelin (SM)/cholesterol (Chol) (PC/SM/Chol, 17/50/33), PC/SM/Chol/brain cerebroside sulfatide (CerSul) (17/45/33/5), PC/SM/Chol/galactosylceramide (GalCer) (17/45/33/5), and PC/SM/Chol/Gb3 (17/45/33/5). A significant and reproducible binding of Hsp70 to unilamellar glycosphingolipid vesicles containing Gb3 was observed (Figure 6). Although a weak interaction of Hsp70 with PC/SM/Chol/GalCer (17/45/33/5) vesicles was also apparent, these studies further define the lipid composition which enables Hsp70 to interact with the plasma membrane of tumor cells and strengthen the concept that Gb3 plays an important role in this interaction process.

## Discussion

Heat shock proteins with a molecular weight of approximately 70 kDa are located in most subcellular compartments and in these they fulfill chaperoning tasks including the folding of newly synthesized polypeptides and their transport across membranes [1], [2]. Although lacking a classical consensual transmembrane sequence, Hsp70, the major stress-inducible member of the HSP70 family, has also been found to be present on the plasma membrane [3], [4], [19] and in the extracellular milieu of cells with an intact membrane [20]–[22]. Membrane localization of HSPs appears to be restricted to transformed [3], [4] and virally infected cells [23], whereas in normal cells, except spermatogenic cells, Hsp70 is strictly localized within the cell. The presence of Hsp70 proteins in the supernatants of viable tumor cells [9], [24], [25] is currently explained by an alternative lysosomal/endosomal pathway, which does not involve the classical ER Golgi compartment [26]. These findings concur with those from Asea and colleagues who demonstrated that drugs which perturb ER Golgi transport, including monensin and brefeldin A, do not influence the release of Hsp70 [27].

The export of Hsp70 requires an interaction of the cytosolic protein with components of the plasma membrane. Given that high salt and major changes in pH fail to eliminate Hsp70 from the plasma membrane of tumor cells (unpublished observations), an electrostatically-driven binding of Hsp70 to protein-based membrane components appears to be unlikely. Rather, it is likely that Hsp70 interacts with fatty acids of the plasma membrane, as has been proposed by Hightower and Guidon in 1989 [7]. However, the molecular nature of the interacting lipid component was not elucidated at that time.

Previous studies have shown that members of the HSP70 family preferentially interact with artificial liposomes in the presence of phosphatidylserine (PS) [28], [29]. If PS serves as the natural binding partner for Hsp70 *in vivo*, then a higher PS content would be expected in Hsp70 membrane-positive tumor sublines. The presence of PS at the outer membrane leaflet, as determined by a specific cell surface staining using the Ca^2+^-dependent phospholipid binding protein Annexin-V [30] serves as an early marker for apoptotic cell death [31]. Since we found here that Hsp70 membrane-positive tumor sublines do not bind Annexin-V-FITC and since cell viability, plating efficiency, and doubling-time was comparable in Hsp70 membrane-positive and -negative tumor sublines, it appears unlikely that PS serves as the interacting partner for Hsp70 in the plasma membrane of the cells used in the current study.

Many reports show that Hsp70 is located in CRMs [8]–[10], which are defined as regions within the plasma membrane that are enriched in cholesterol, glycosphingolipids, glycosylphosphatidylinositol-anchored proteins and some other acetylated proteins [32]. CRMs, also termed as lipid rafts [33], serve as assembly and sorting platforms for signal transduction complexes, increase cell interactions and thus enhance inter-cellular crosstalk. These functions might explain the presence of stress proteins with chaperoning function in CRMs. In line with previous findings from others, cholesterol depletion with MbCD results in a loss of Hsp70 from the plasma membrane of tumor cells.

A comparative lipidomic analysis of the content of CRM-residing glycosphingolipids revealed significantly greater amounts of globotriaosylceramide Gb3 [34] in Hsp70 membrane-positive tumor sublines, as compared to their Hsp70 membrane-negative counterparts. In contrast, the content of other ceramide-derived glycosphingolipids such as lactosylceramide, dodecasaccharideceramide, galactosylcereamide and ceramide in Hsp70 membrane-positive and -negative tumor sublines were similar. Gb3 is known to serve as a receptor for Shiga toxin, an enterotoxin produced by *Shigella dysenteria* and enterohemorrhagic *Escherichia coli*, and is frequently found on germinal centre B cells and on tumor cells [35]–[37], but rarely on normal cells. The selective enrichment of Gb3 in the plasma membrane of Hsp70-positive tumor sublines led us to the hypothesis that Gb3 might act as an interaction partner for Hsp70 in the plasma membrane. In line with these results we could further show that Hsp70 was eliminated from the plasma membrane concomitant with a loss of ceramide-based glycosphingolipids, including Gb3, following treatment with PPMP1. Previous work of Lingwood et al has demonstrated that Hsp70 binds to 3′-sulfogalactolipids via the ATPase domain [38]. Based on binding patterns of Hsp70 antibodies that detect different epitopes in the ATPase and the C-terminal substrate binding domain, the orientation of Hsp70 in Gb3 containing CRMs appears to be identical (unpublished observation). Together with the finding that Hsp70 interacts only with artificial raft-like liposomes containing Gb3, we conclude that Gb3 has an important role in the interaction of Hsp70 with the plasma membranes of Hsp70 membrane-positive tumors.

## Materials and Methods

### Chemicals and Reagents

All chemicals and reagents were obtained from Sigma-Aldrich or from Carl Roth unless stated otherwise.

### Cells and Cell Culture

Established human colon (CX2; Tumorzellbank, DKFZ, Heidelberg, Germany) and pancreas (Colo357; Centre for Applied Microbiology and Research, Salisbury, UK) carcinoma cells were separated into the sublines CX+/CX− and Colo+/Colo− by fluorescence activated cell sorting using the FITC-conjugated Hsp70-specific mAb cmHsp70.1 (multimmune GmbH, Munich, Germany). CX+ (87%) and Colo+ (73%) tumor sublines contain consistently high proportions of Hsp70 membrane-positive cells, and CX− (23%) and Colo− (34%) contain consistently low proportions of Hsp70 membrane-positive cells [16], [17]. The human cervix carcinoma cell line HeLa was stably transfected with an expression vector harboring mouse Bag-4 cDNA (pcDNA-SODD, access code Q8CI61), or with the corresponding control vector, neo. DNA (8 µg) was electroporated into 5×10^6^ cells using a BioRad gene pulser (750 V, 25 µF, 200 Ohm, 0.4 cm cuvettes) and cells were selected in medium containing G418 (0.3 mg/ml, Calbiochem, San Diego, USA). Bag-4 stably transfected clones overexpressing Hsp70 on their cell surface were selected and cultured as described above [18]. Plating efficiency, doubling time (20 h), and protein content (indicated in Figure 2A) in membrane Hsp70 low- and high-expressing tumor sublines were similar.

The mycoplasma-free human erythroleukemic (K562) and Burkitt's lymphoma (Daudi) tumor cell lines and primary fibroblasts derived from a patient with Fabry disease having a high Gb3 content were maintained in exponential growth by regular passages (typically every 3 days) in RPMI 1640 supplemented with 5% v/v heat-inactivated fetal calf serum (FCS), 50 IU/ml penicillin, 50 µg/ml streptomycin, 2 mM L-glutamine, and 1 mM sodium pyruvate (Life Technologies, Eggenstein, Germany).

### Biotinylation of Membrane Proteins, SDS-PAGE and Western Blot Analysis

Following three washing steps in ice cold, serum-free phosphate buffered saline (PBS, pH 7.5), viable tumor cells (>97%) were resuspended in PBS at a density of 1×10^6^ cells/ml and incubated with biotinylation reagent (EZ-Link sulfo-NHS-LC-biotin, 1 mg/ml, Pierce) under gentle rotation. After 30 min, the biotinylation process was terminated by the addition of Tris-HCl (pH 7.5) to a final concentration of 50 mM. After 3 washing steps in PBS, cells were solubilized in lysis buffer (1% v/v Triton X-100) containing protease inhibitors and completely disrupted by dounce homogenization (20 strokes). Solubilized biotinylated membrane proteins were centrifuged (14,000 rpm for 30 min at 4°C) and supernatants were purified twice on ImmunoPure (Pierce) avidin columns according to the manufacturer's recommended protocol. Biotinylated membrane proteins were eluted from the column with 5 mM biotin in PBS containing 1% v/v Triton-X, concentrated on Centricon YM-3 columns (Millipore) and subjected to SDS-PAGE.

For SDS-PAGE, solutions of biotinylated membrane proteins and whole cell membranes were denatured in sample buffer (25 mM Tris hydrochloride (pH 6.8), 2% w/v SDS, 10% v/v glycerol, 10% v/v dithiothreitol, 0.2% w/v bromophenol blue) and heated for 10 min at 95°C. Proteins were separated by 10% SDS-PAGE under reducing conditions [39], blotted onto nitrocellulose membranes [40] and stained either with Ponceau S solution or primary mAbs directed against Hsp70 (cmHsp70.1, multimmune GmbH or SPA-810, Assay Designs, Ann Arbor, USA), ganglioside GM1 or tubulin. Biotinylated proteins and bound mAbs were visualized using a horseradish peroxidase complex or peroxidase-conjugated secondary antibodies (Dako, Hamburg, Germany) and chemiluminescence (ECL, Amersham Biosciences, Chalfont St. Giles, UK). The amount of protein in the samples was quantified by densitometry and compared to the signals generated by tubulin staining and corresponding recombinant Hsp70 protein (NSP-555, Assay Designs) which was run in parallel (100 ng per lane).

### Analysis of Lipid Composition

Plasma membranes and CRMs were isolated as mentioned above and analyzed by mass spectrometry, as described elsewhere. Briefly, lipids were quantified by electrospray ionization tandem mass spectrometry (ESI-MS/MS) in positive ion mode as described previously [41]–[43]. Samples were quantified by direct flow injection analysis using the analytical setup described by Liebisch et al. [42], [43]. A Precursor Ion Scan of m/z 184 specific for phosphocholine containing lipids was used for phosphatidylcholine (PC) and sphingomyelin (SM) [42]. Neutral Loss Scans of m/z 141 and m/z 185 were used for phosphatidylethanolamine (PE) and phosphatidylserine (PS) respectively [41]. Ceramide was analyzed using a similar approach to that described previously [44] using N-heptanoyl-sphingosine as an internal standard. Free cholesterol (FC) and cholesteryl ester (CE) were quantified using a fragment ion of m/z 369 after selective derivatization of FC using acetyl chloride [42]. Correction of isotopic overlap of lipid species as well as data analysis by self programmed Excel Macros was performed for all lipid classes according to previously described principles [43].

### Methyl-Beta Cyclodextrin (MbCD) and 1 Phenyl-2-Hexadecanoyl Amino-3-Morpholino-1-Propanol (PPMP1) Treatment

Tumor cells were incubated with 1 mM and 10 mM of the raft-disrupting agents MbCD, for 1 h at 37°C (Sigma-Aldrich), washed and then resuspended in fresh medium. Tumor cells were also treated with a sublethal dose of PPMP1 (25 µM) for 3 or 6 days in order to eliminate ceramide-based glycosphingolipids, including Gb3, from the plasma membrane.

### Flow Cytometry

Following a washing step, untreated and cholesterol-depleted viable tumor cells were counted and 0.1×10^6^ cells were incubated with an FITC-conjugated cmHsp70.1 mAb, and antibodies directed against Gb3 (CD77, BD Biosciences, Heidelberg, Germany; [Gal(α1–4)Gal(β1–4)Glc ceramide]), LacCer (lactosylceramide, CDw17, BD Biosciences), DoCer (dodecasaccharideceramide, CD65s, BD), GalCer (galactosylceramide, MAB 342, Chemicon International, Temecula, USA), Cer (ceramide, MID15B4, Axxora Life Sciences, San Diego, USA), a secondary PE-conjugated antibody (Dako) or an FITC-conjugated IgG1 isotype-matched control (Cymbus Biotechnology, Eastleigh, UK), for 15 min at 4°C. GM1 was visualized by incubating the tumor cells with 5 µl of the Alexa Fluor 555-conjugated cholera toxin subunit B (dilution 1∶10, Molecular Probes Europe BV, Leiden, Netherlands) for 30 min at room temperature. Cells were washed and resuspended in PBS containing 10% v/v heat-inactivated FCS. Only viable, 7-amino-actinomycin D negative (7-AAD, BD Biosciences) cells were analyzed on a FACSCalibur™ flow cytometer (BD Biosciences). To determine the PS content in the outer leaflet, cells were incubated with 5 µl Annexin-V-FITC in 150 µl Ca^2+^ containing Annexin-V-binding buffer (BD Pharmingen) for 15 min at room temperature in the dark. Mean fluorescence intensities (mfi) were determined by subtracting the value obtained using the isotype-matched control immunoglobulin from the experimental value.

### Immunostaining

Tumor cells were stained either with 2 µl of the FITC-conjugated Hsp70-specific mAb cmHsp70.1 (0.5 µg/µl), 5 µl of the Alexa Fluor 555-conjugated cholera toxin B subunit (dilution 1∶10, Molecular Probes Europe BV), Gb3/CD77 antibody (BD Biosciences) and a secondary Cy3-conjugated antibody (Dako), filipin III (50 µg/ml; Sigma-Aldrich) which binds cholesterol within the plasma membrane, or with the relevant isotype-matched control antibodies for 30 min in the dark. Cells were then transferred onto glass slides in Fluorescent Mounting Medium (Dako) and cytospin preparations were made. Slides were analyzed by transmission and fluorescence microscopy using a Leica SP5 confocal microscope equipped with ×100 (planar) and ×63 (apochromatic) oil-immersion objective (Acousto Optical Beam Splitter system). Pictures of specifically stained cells and overlays were taken and editing was performed using Metamorph® software (Universal Imaging, Downingtown, USA). The location of cholesterol is visualized in blue (filipin III), that of Hsp70 in green (FITC), and that of Gb3 in red (Cy3). An overlay technique revealed the co-localization of Hsp70 and Gb3 in yellow.

### Protein Binding Assay

Gb3-negative (K562) and Gb3-positive (Daudi) tumor cell lines were incubated either with Hsp70-FITC or BSA-FITC at different concentrations (6–24 µg/ml) for 30 min on ice. After 2 washes in PBS cells were analyzed by flow cytometry, as described above. For blocking assays, the tumor cell lines (1×10^6^) were incubated either with 5 µg/ml of unlabeled Gb3 (CD77) or ceramide (MID15B4) antibodies for 20 min on ice, followed by FITC-Hsp70 (60 µg/ml).

### Vesicle Copellation Assay

Unilamellar vesicles with a nominal diameter of 200 nm were prepared according to the extrusion method. Briefly, mixed lipid films were prepared under a stream of nitrogen followed by 3 h under vacuum and stored at 4°C. Multilamellar vesicles (MLVs) were formed by first swelling the lipid films in buffer solution (50 mM TRIS/HCl, pH 7.4) for 30 min followed by vortexing three times for 30 sec every 5 min. Large unilamellar vesicles (LUVs) were produced form MLVs by pressing them through a polycarbonate membrane with 200 nm pore diameter using a mini extruder (LipoFast, Avestin, Canada). LUVs (1 mg/ml) were incubated with Hsp70 (1 µg/ml) for 30 min at room temperature in 1 mM Bis/Tris buffer, pH 7.4 and then centrifugated at 4°C (200,000× *g*). Pelleted fractions were subjected to SDS-PAGE analysis.
